# Iron Overload in Myelodysplastic Syndromes: Pathophysiology, Consequences, Diagnosis, and Treatment

**Published:** 2018-05-01

**Authors:** Lindsey Lyle, Alex Hirose

**Affiliations:** University of Colorado, Aurora, Colorado

## Abstract

Myelodysplastic syndromes (MDS) are a heterogeneous group of hematologic neoplasms varying in severity affecting one or more lines of hematopoiesis. Ineffective erythropoiesis results in dysregulation of iron metabolism. Most MDS patients have anemia, and some require regular red blood cell transfusions. These transfusions, in addition to factors of the disease itself, can result in iron overload (IO). Retrospective analyses suggest that MDS patients with IO have reduced overall survival and poorer outcomes following allogeneic stem cell transplant vs. those without IO. Iron chelation therapy (ICT; deferoxamine, deferasirox, or deferiprone) has been used to alleviate IO in other transfusion-dependent hematologic conditions (e.g., thalassemia), but its role in MDS has not been firmly established. A growing body of evidence suggests that ICT in MDS patients is an effective means for reducing transfusional IO and may significantly improve outcomes such as survival. The orally administered iron chelator deferasirox has been widely studied in MDS, and available studies have shown it to be generally well tolerated and effective in reducing IO in this population. The pathophysiology and clinical consequences of IO in MDS, as well as current methods for diagnosing and treating IO in these patients, are discussed.

Myelodysplastic syndromes (MDS) encompass a heterogeneous group of hematologic malignancies that are characterized by ineffective hematopoiesis and risk for progression to acute myeloid leukemia (AML; Mitchell, Gore, & Zeidan, 2013; Petrou et al., 2015; Shah, Kurtin, Arnold, Lindroos-Kolqvist, & Tinsley, 2012). The majority of patients with MDS (approximately 80%) are anemic, and a large percentage of them will require red blood cell (RBC) transfusional support during their disease course (Shenoy, Vallumsetla, Rachmilewitz, Verma, Ginzburg, 2014; Temraz, Santini, Musallam, & Taher, 2014). As a result of ineffective erythropoiesis and continued transfusion dependence, MDS patients are prone to excessive iron accumulation and, ultimately, iron overload (IO; Shah et al., 2012; Shenoy et al., 2014). Iron overload in MDS patients may cause organ deposition of excess iron, resulting in endocrinopathies, liver, and cardiac dysfunction. Accordingly, retrospective and observational studies have suggested patients with MDS and IO have a markedly increased risk of death compared with MDS patients without IO (Adams & Bird, 2013; Mitchell et al., 2013; Shah et al., 2012; Steensma & Gattermann, 2013; Wood, 2015). Iron overload in MDS patients may also be a risk factor leading to poor outcomes following allogeneic stem cell transplant (ASCT; Jacobi & Herich, 2016; Wermke et al., 2012).

The current methods for evaluating IO range from the simple and readily available assessments of plasma markers such as serum ferritin (SF) to more sophisticated approaches involving magnetic resonance imaging (MRI; Wood, 2015). Alleviation of IO using iron chelation therapy (ICT) with iron chelators has been suggested as a means to prevent end-organ complications and improve survival, although prospective data and evidence-based guidance on the use of ICT in MDS are still lacking (Mitchell et al., 2013). The purpose of this article is to review the pathophysiology of IO in MDS, the clinical consequences of IO, current methods for diagnosing IO in MDS, and the treatment of IO using ICT.

## IRON OVERLOAD IN MDS: PATHOPHYSIOLOGY AND CONTRIBUTING FACTORS

**Normal Iron Homeostasis**

**Figure 1:**
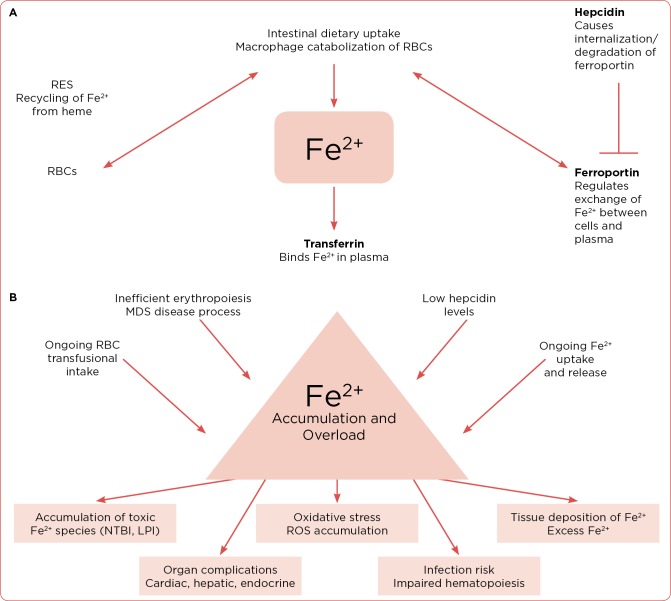
(A) Regulation of iron homeostasis. (B) Development of iron overload in patients with myelodysplastic syndromes. RES = reticuloendothelial system; RBC = red blood cell; MDS = myelodysplastic syndromes; NTBI = non–transferrin bound iron; LPI = labile plasma iron; ROS = reactive oxygen species.

Under normal homeostatic conditions, levels of iron in the body must be tightly regulated, as iron becomes toxic when present in excess, and there is no physiologic mechanism for iron excretion (Adams & Bird, 2013; Mitchell et al., 2013; Shah et al., 2012; Shenoy et al., 2014; Steensma & Gattermann, 2013; Wood, 2015). The principal storage of dietary iron consists of RBCs (~60%) and SF (~25%), as well as heme enzymes (e.g., cytochromes, catalases, peroxidases) and nonheme enzymes (e.g., ribonucleotide reductase; Shah et al., 2012). When RBCs become senescent and die, their hemoglobin is catabolized by macrophages of the reticuloendothelial system (RES) and the iron is recycled. Dietary iron is also taken up by intestinal cells, and this dietary uptake as well as iron recycling by macrophages is modulated by the iron transporter ferroportin (Figure 1A).

Some of the principal molecules involved in iron homeostasis include transferrin, hepcidin, and ferroportin. Transferrin binds to excess iron and serves as the principal transporter of free iron in the blood; transferrin binds to the transferrin receptor, which has limited binding capacity, and toxic iron species may accumulate at ~75% transferrin saturation (Kim & Nemeth, 2015; Merkel & Nagler, 2014; Porter, de Witte, Cappellini, & Gattermann, 2016; Sebastiani, Wilkinson, & Pantopoulos, 2016; Shah et al., 2012). Ferritin is the principal protein involved in the storage of intracellular iron (Merkel & Nagler, 2014; Shah et al., 2012). Hepcidin is produced by the liver and regulates the intestinal uptake of iron from the diet and release of iron from macrophages of the RES. Levels of hepcidin are regulated by iron concentrations and erythropoiesis, and abnormal hepcidin production can lead to significant disruptions in iron homeostasis (Kim & Nemeth, 2015; Sebastiani et al., 2016). Ferroportin is the membrane iron exchange transporter molecule present on enterocytes, macrophages, and hepatocytes, and serves as a principal regulator of dietary iron absorption, recycling via the RES, and cellular iron storage in the liver, respectively (Kim & Nemeth, 2015; Shah et al., 2012).

**Impact of MDS Disease Process**

Disruptions in hepcidin levels can have a dramatic impact on iron homeostasis. Ineffective erythropoiesis, a hallmark of MDS, causes a massive expansion of bone marrow erythroblasts due to decreased production of mature RBCs (Sebastiani et al., 2016). This creates a high demand for iron, leading to the suppression of hepcidin. In one study, mean hepcidin levels were found to be consistently heterogeneous across different MDS subtypes (n = 113), likely reflective of their different clinical and pathologic features (Santini et al., 2011). Whereas the highest levels were observed in refractory anemia with excess blasts (RAEB; 11.3 nm) or in chronic myelomonocytic leukemia (CMML; 10.04 nm), the lowest hepcidin levels were seen in refractory anemia with ringed sideroblasts (RARS; 1.43 nm; *p* = .003 by analysis of variance [ANOVA]). Consistent with the mechanism of action of hepcidin, RARS patients in this study tended to have the highest levels of toxic non–transferrin bound iron (NTBI; 1.59 µM) while levels in the RAEB and CMML patients were lower (0.03 and 0.19 µM, respectively; *p* = .058; Santini et al., 2011). Thus, patients with MDS are at risk for IO prior to their becoming transfusion dependent, due to the disease processes seen in MDS, which results in reduced hepcidin production by the liver and increased iron absorption from the gut, ultimately leading to IO (Figure 1B; Chaudhary & Pullarkat, 2013; Sebastiani et al., 2016; Steensma & Gattermann, 2013). This is an important observation, as most of the current concerns in MDS patients may be focused exclusively on transfusion dependence and less so on the disease process.

In one study of 107 MDS patients in China who had not received prior transfusions, the investigators found that SF levels were elevated, serum hepcidin levels were elevated, and the hepcidin to ferritin ratio was significantly decreased (*p* < .001) relative to control individuals with a normal iron state (Cui et al., 2014). There was also a negative correlation between the hepcidin to ferritin ratio (a measure of adequacy of hepcidin levels, relative to body iron stores) and erythropoietin levels (*r* = –0.449; *p* < .001) in this study.

The findings suggest that in MDS patients, serum hepcidin levels are inappropriately low, and the degree of hepcidin response is blunted relative to normal. Specifically, in this study, the authors hypothesize that tissue hypoxia, resulting from ineffective erythropoiesis, triggers increased erythropoietin production, which then results in low hepcidin; the inappropriately low hepcidin then causes an increase in iron absorption and release from storage, causing saturation of transferrin and resulting in IO (Cui et al., 2014). In view of the essential role of hepcidin in iron homeostasis, there is also evidence that using ICT may help normalize hepcidin levels in MDS. In one study of 19 MDS patients, serum hepcidin increased following 12 weeks of ICT with deferasirox (Exjade, Jadenu; Ghoti et al., 2011).

**Impact of Genetic Factors**

*SF3B1* is a gene encoding a component of the RNA splicing machinery, and mutations in this gene have been found in MDS patients with RARS; in one study, a mutation in *SF3B1* was found in 28% of MDS patients (N = 76), and the proportion of mutation in RARS patients (55%) was higher than that of other MDS categories (9%; *p* < .001; Ambaglio et al., 2013). There was a significantly lower hepcidin to ferritin ratio in those with *SF3B1* mutation as compared with those without the mutation (*p* = .004), and there was a significant relationship between hepcidin to ferritin ratio and *SF3B1* mutation burden (*r* = –0.37; *p* = .001; Ambaglio et al., 2013). The results suggest that expanded but ineffective erythropoietic activity in these patients leads to inappropriately low hepcidin levels in MDS-RARS patients with an *SF3B1* mutation, leading ultimately to excess release of iron from the RES and parenchymal iron loading (Ambaglio et al., 2013).

Another more recent study observed *SF3B1* mutations in 63% (33 of 52) of patients with RARS; frequency was highest among patients with RARS (83%) relative to other World Health Organization (WHO) morphologic categories (Zhu et al., 2016). As compared with the wild type (WT) RARS patients, there was a more severe IO (higher SF, lower hepcidin) and over-erythropoiesis (more active marrow function and ineffective erythropoiesis) in those with *SF3B1* mutations; however, there was better survival in the *SF3B1*-mutant patients, largely due to their lower International Prognostic Scoring System (IPSS) risk categorization (low-risk IPSS ≤ 1.0, 94% vs. 58% for mutant vs. WT, respectively). There was also a higher incidence of other gene mutations (e.g., *ASXL1*, *SRSF2*, *U2AF1*) in the *SF3B1* WT group (80%) as compared with the mutant group (39%; Zhu et al., 2016).

**Impact of Transfusional Dependence**

Red blood cell transfusions are the main source of progressive IO in transfusion-dependent patients, adding to the already dysregulated iron homeostasis as a function of MDS disease processes (Shenoy et al., 2014; Steensma & Gattermann, 2013). Each unit of transfused blood delivers an additional 200 to 250 mg of iron (Adams & Bird, 2013; Shah et al., 2012; Steensma & Gattermann, 2013). The net excretion of iron daily is only about 1 to 2 mg/day, and this loss is generally replenished by dietary intake. The approximate iron absorption in a healthy patient is 4 mg in 1 year (Steensma & Gattermann, 2013). An MDS patient with a requirement of 4 RBC units per month accumulates approximately 9.6 g of iron per year, which is a nearly 6-fold higher yearly iron burden (Shenoy et al., 2014). Over a 2-year period, approximately 20 g of iron will accumulate, whereas the total body storage of iron is typically under 4 g (Steensma & Gattermann, 2013). It is known from patients with hereditary hemochromatosis that clinical manifestations of IO will typically occur at concentrations over 15 to 20 g.

## CONSEQUENCES OF IRON OVERLOAD IN MDS PATIENTS

Whereas excess iron accumulated through intestinal absorption is usually stored in the liver, the liver, myocardium, and endocrine organs are most susceptible to damage with continued iron loading (Porter et al., 2016; Steensma & Gattermann, 2013; Temraz et al., 2014). Accumulation of toxic iron species such as NTBI and labile plasma iron (LPI) may also occur, resulting in increased levels of reactive oxygen species (ROS) and subsequent tissue damage (Figure 1B; Porter et al., 2016). Mechanisms are incompletely understood, but IO can also inhibit erythropoiesis, and, accordingly, ICT has been shown to improve hemoglobin levels and reduce transfusion requirements, which will be discussed in the following pages (Shenoy et al., 2014). Although the consequences of transfusional IO such as cardiac disease, endocrine disturbances, and liver fibrosis/cirrhosis are best described in thalassemia major and related inherited anemias, they are increasingly recognized in MDS (Porter et al., 2016).

Iron overload–related hepatic and cardiac dysfunction and endocrinopathies (Figure 1B) may contribute to an increased risk of death in patients with MDS (Adams & Bird, 2013; Mitchell et al., 2013; Shah et al., 2012; Steensma & Gattermann, 2013; Wood, 2015). Infection risk may also be increased in MDS patients with IO due to the direct effects of iron on bacterial and/or fungal growth and through immune impairment (Toma, Fenaux, Dreyfux, & Cordonnier, 2012). Although infections are typically attributable to neutropenia in MDS patients, those with IO may also have reductions in key immune cytokines (e.g., TNFα, IFNγ), impaired nitric oxide production, and loss of T-cell function, which can further contribute to infection risk (Toma et al., 2012).

In a study of patients undergoing ASCT, the SF level of 1,000 ng/mL or greater was associated with a significantly increased risk of infection (hazard ratio [HR], 2.87; *p* = .003), proven fungal infection (HR, 4.04; *p* = .001), and worsened survival in univariate analysis (HR, 2.09; *p* = .033; Jacobi & Herich, 2016). Reactive oxygen species and oxidative stress associated with IO may also be detrimental to bone marrow progenitors, and ICT may help to alleviate oxidative stress associated with IO. In experimental studies, IO has been shown to have inhibitory effects on hematopoiesis, affecting the function of hematopoietic stem and progenitor cells and reducing the number of hematopoietic stem cells. This is thought to be related to the upregulated NOX4/ROS/P38 MAPK signaling pathways suggesting IO-induced chronic oxidative stress in hematopoietic stem and progenitor cells (Chai et al., 2015).

There is growing evidence that IO may adversely impact outcomes following ASCT (Armand et al., 2011). One study showed that systemic IO defined in the study as liver iron concentration (LIC) level of 125 µmol or greater (as assessed by MRI) was a predictive factor associated with nonrelapse mortality (NRM) in patients with AML or MDS undergoing ASCT; patients with systemic IO in this study had a higher cumulative incidence of NRM in the first 100 days after transplant vs. those without (by day 100, 27.3% vs. 4.7%, respectively), with most patients succumbing to infection or graft-vs.-host disease (Wermke et al., 2012). In a recent study of patients undergoing ASCT (N = 201), transfusion dependence prior to transplant was shown to have a negative prognostic impact on outcomes following ASCT; this included overall survival (HR, 1.99; *p* = .006), nonrelapse mortality (HR, 1.89; *p* = .03), and relapse incidence (HR, 2.67; *p* = .03; Cremers et al., 2016). Multivariate analysis showed a significantly decreased overall survival (OS) in patients who received more than 20 RBC transfusions prior to conditioning (HR, 1.99; *p* = .006). In a study of 48 transfusion-dependent patients undergoing ASCT (17% MDS patients), 85% of patients were found to have hepatic IO (defined as LIC above the upper limit of normal) and 42% had significant hepatic IO (defined as LIC ≥ 5.0 mg/g dry weight [dw]; Armand et al., 2011).

**Figure 2:**
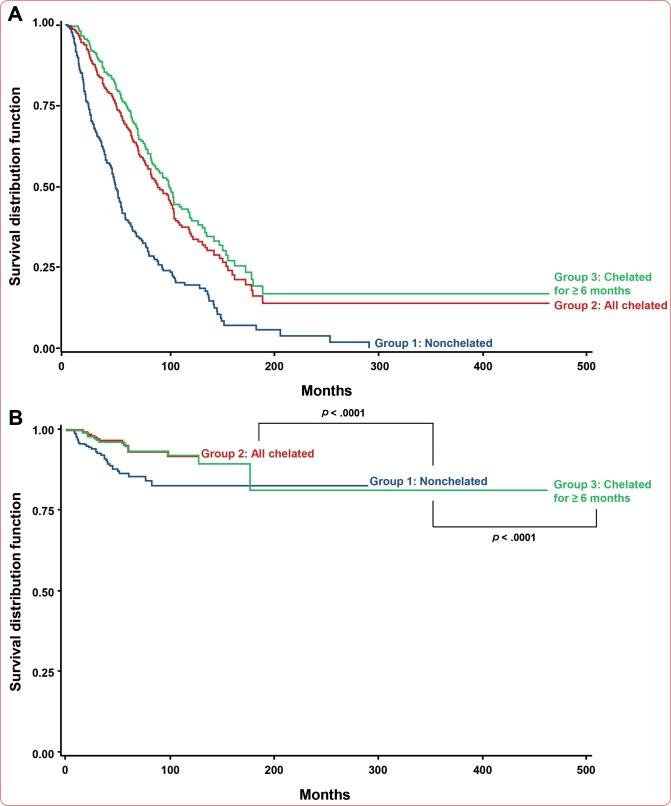
(A) Overall survival for all enrolled patients in the US 22 study evaluating the impact of chelation on overall survival in iron overloaded patients with lower-risk MDS. (B) Progression to acute myeloid leukemia for all enrolled patients in the US 22 study. Reprinted from Lyons et al., Relation Between Chelation and Clinical Outcomes in Lower-Risk Patients With Myelodysplastic Syndromes: Registry Analysis at 5 Years, Leukemia Research, 56, 88–95, Copyright © 2017, with permission from Elsevier.

Studies have also established a deleterious role for IO in low-risk MDS patients not undergoing transplant and a beneficial role of ICT in this population (Remacha et al., 2015). A recent Polish study showed that transfusion dependence and hyperferritinemia (> 1,000 ng/mL) in MDS patients were associated with shortened median survival (320 days vs. 568 days; *p* = .014), and the authors suggest that these may serve as negative prognostic factors for MDS patients (Waszczuk-Gajda et al., 2016). In one Spanish study (N = 263), the patient’s age (*p* = .011), IPSS (*p* < .001), and chelation treatment (*p* = .015) were predictors for overall survival, and leukemia-free survival was predicted by IPSS (*p* = .014) and transfusion frequency (*p* = .001; Remacha et al., 2015). In another registry study of iron overloaded (SF ≥ 1,000 ng/mL) MDS patients (N = 3,552, diagnosed between 1975 and 2008), OS was higher in chelated vs. nonchelated (supportive care only) patients (75 vs. 49 months; *p* = .002), but no difference in AML transformation was seen (*p* = .73; Neukirchen et al., 2012). The US 22 study was a 5-year observational prospective registry study that enrolled 600 low-risk MDS patients with IO recruited from 118 centers. Results from the study showed a significant benefit in survival for the chelated patients and the patients chelated more than or equal to 6 months relative to the nonchelated patients (Figure 2A) with a median time to death from MDS diagnosis of 86.3, 98.7, and 47.8 months, respectively (*p* < .0001; Lyons et al., 2017). There was a reported significant benefit in terms of time to progression to AML for the chelated patients (Figure 2B), with a median time from diagnosis to leukemic progression of 86.3, 97.8, and 46.7 months in the respective groups (*p* < .0001; Lyons et al., 2017). The observed benefit of ICT in reducing progression to AML in this study is intriguing and will require further study.

## CURRENT METHODS FOR DIAGNOSIS OF IRON OVERLOAD

**Figure 3:**
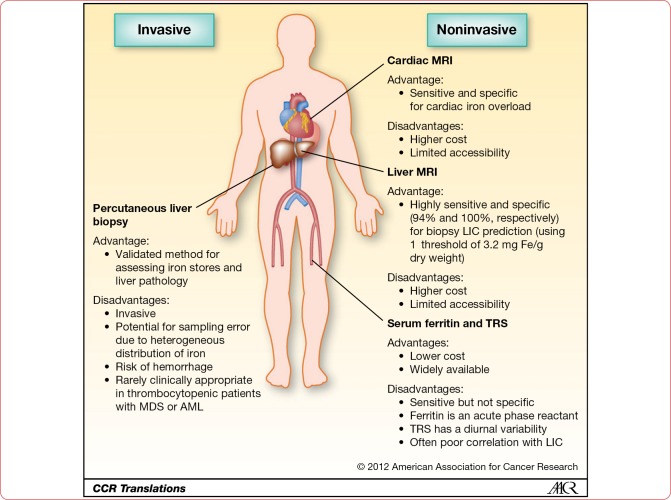
Advantages and disadvantages of different modalities to assess iron overload. MRI = magnetic resonance imaging; MDS = myelodysplastic syndromes; AML = acute myeloid leukemia; LIC = liver iron concentration; TRS = transferrin saturation. Reprinted from Kruger, Leahy, & Olynyk, Assessing Iron Overload: Are We There Yet?, Clinical Cancer Research, 2012, 18(23), 6395–6397, with permission from AACR.

The benefits and disadvantages of some of the currently available methods for IO assessment in MDS patients are summarized in Figure 3 (Kruger, Leahy, & Olynyk, 2012). Serum ferritin is a rapid, widely available, and low-cost option to assess IO in MDS patients (Adams & Bird, 2013). A disadvantage is that SF is an acute phase protein; therefore, SF levels may also be impacted by events such as tissue injury and inflammation. Despite this, SF has been a frequent assessment in clinical trials, and an SF level of ≥ 1,000 ng/mL has recently been validated as a marker of IO and poor prognosis following ASCT (Jacobi & Herich, 2016).

In this study, iron levels as measured by a superconducting quantum interference device (SQUID) correlated highly with SF levels (*r* = .6; *p* < .001), and SF was predictive of worse overall and event-free survival (Jacobi & Herich, 2016). Another study suggested that SF is a reasonable surrogate to detect IO in patients undergoing ASCT, although the authors propose increasing the threshold to 2,500 ng/mL (Armand et al., 2011). As noted earlier, LIC as assessed via MRI was associated with NRM in AML or MDS patients undergoing ASCT (Wermke et al., 2012). By comparison, an SF level of 1,000 ng/mL or even as high as 2,500 ng/mL was not associated with NRM in this study, and the authors suggest that LIC, and not SF (which can be impacted by conditions such as inflammation), should be used to stratify risk in MDS patients undergoing ASCT (Wermke et al., 2012). Other equally accessible serum markers such as LPI and NTBI are also being investigated as potential markers of IO in MDS patients, and these may be a better indicator of the levels of actual toxic iron species in the body that can result in organ damage (de Swart et al., 2016; Gu et al., 2017). Nevertheless, rigorous validation and standardization of the available NTBI and LPI assays, a consensus of how to report results, and the establishment of toxic thresholds is needed before these measures of IO can be routinely used for treatment decisions in clinical practice (de Swart et al., 2016).

Magnetic resonance imaging of the liver (e.g., liver R2 or R2*), heart (e.g., cardiac T2*), or endocrine glands (e.g., pancreas R2*) is also increasingly being investigated as a means to evaluate IO in MDS patients (Petrou et al., 2015; Wood, 2015). For example, the liver is a central organ for the storage of excess iron in the body, and MRI-based estimates of LIC have become the gold standard for assessing IO (Armand et al., 2011; Merkel & Nagler, 2014). However, a meta-analysis evaluating the MRI concluded that studies using a more rigorous design, with explicit and standardized MRI thresholds for IO are needed to establish its role as a surveillance tool; it may also not be pragmatic or feasible to evaluate LIC using MRI in all patients (Sarigianni et al., 2015). Similarly, the use of regular MRI cardiac T2* monitoring has also been proposed (Pascal et al., 2013), although not all authors agree there is sufficient evidence for a monitoring benefit (Bowen, Hellström-Lindberg, & Steensma, 2014), and it is also subject to the limitations of MRI accessibility, feasibility, and costs.

## USE OF IRON CHELATION THERAPY IN MDS

Results of a meta-analysis of eight observational studies demonstrated that the use of ICT in low-risk MDS patients resulted in better median survival time as compared with those not using ICT (Mainous, Tanner, Hulihan, Amaya, & Coates, 2014). Currently available chelation agents include deferoxamine (Desferal), deferiprone (Ferriprox), and deferasirox (Merkel & Nagler, 2014; Taran & Taran, 2015; Temraz et al., 2014). Due to the limitations of deferoxamine (e.g., requirement for parenteral administration, poor patient adherence) and deferiprone (possibility for agranulocytosis), deferasirox has been considered a first-line ICT choice in MDS (Santini et al., 2010). Comparative studies between deferasirox and deferiprone have also indicated better reduction in SF with deferasirox, and it has been suggested that deferiprone may be more appropriate for patients with a low IO burden or in whom deferasirox is not tolerated (Cermak et al., 2013).

There is also evidence to suggest that deferasirox, unlike deferiprone, has actions independent of its iron-chelating property, which modulates ROS, and may influence key factors involved in self-renewal and/or the differentiation of hematopoietic stem or progenitor cells; these effects could underlie the beneficial effects on hematopoiesis that have been observed in MDS patients (Tataranni et al., 2015). The Evaluation of Patients’ Iron Chelation with Exjade (EPIC) was the largest assessment of ICT with deferasirox in an MDS cohort, consisting of some 1,744 patients, 341 of whom had MDS with a median SF value at baseline of 2,730 ng/mL (Shenoy et al., 2014). The results of the study showed a significant reduction in SF from baseline (–264 ng/mL; *p* < .0001), and this reduction was reflective of a dose adjustment of deferasirox and ongoing iron intake during the trial (Cappellini et al., 2010). The discontinuation rate in the MDS patients, however, was 48.7%, and drug-related adverse events (AEs) were more common in the MDS group (66.7%); the most common AEs were diarrhea and rash, and these were the most common AEs leading to discontinuation overall (Cappellini et al., 2010). Another open-label, single-arm, phase III trial (US03) included 176 MDS patients; in this study, a reduction in SF of over 36% was observed with treatment beyond 2 years, even with a continued transfusion requirement (List et al., 2012).

Notably, hematologic parameters also improved in 15% to 22% of patients over 1 year of deferasirox treatment in the US03 trial, an important finding as reduction in transfusion requirement is clinically relevant. Similar results were seen in a prospective, open-label, single-arm, multicenter trial in transfusion-dependent patients with low-risk MDS (Angelucci et al., 2014), and the apparent hematologic benefits of deferasirox chelation therapy have been summarized across six European and US trials, encompassing some 760 MDS patients with transfusion-related IO (Breccia et al., 2015). The findings show improvements in hemoglobin in up to 44.5% (41 of 92 evaluable patients), increases in platelet count in up to 61% (8 of 13 patients), and up to 76% of patients (13 of 17 patients) with improvement in neutrophil counts (Breccia et al., 2015). The mechanisms involved are as of yet unknown, but appear to be unrelated to changes in markers such as SF.

Results of these and other large trials and longer-term studies have supported the safety and efficacy of deferasirox in selected and unselected MDS populations (Gattermann et al., 2011, 2012; Improta et al., 2013). Hematologic and hepatic function were also improved in MDS or aplastic anemia patients treated with deferasirox with reductions in SF and LIC (Cheong et al., 2014). Results from routinely treated, nonselected MDS patient populations in real-world settings also showed that deferasirox was active and safe in iron-overloaded patients regardless of previous chelation treatment (Breccia et al., 2012; Maurillo et al., 2015). In a multicenter retrospective "real-world" study of transfusion-dependent MDS patients (N = 118) at 11 centers in Italy, deferasirox reduced median SF levels from 1,790 ng/mL at baseline to 1,689 ng/mL (6 months) to 1,304 ng/mL (12 months), and to 1,140 ng/mL by 2 years, and importantly, the reduction was statistically significant beginning at 6 months onward (*p* < .001). Transfusion independence was also achieved by 6 patients (7.1%), and erythroid (17.6%), platelet (5.9%), and neutrophil (7.1%) hematologic improvements were also seen between months 6 and 24 of deferasirox treatment (Maurillo et al., 2015).

Assessment of efficacy in most deferasirox studies has typically been via SF; however, results of a 1-year, phase II, open-label, multicenter, single-arm study have also shown a reduction in IO as assessed via LIC (using R2 MRI methods; Kohgo et al., 2015). Patients in the study (N = 102) had MDS or other anemias and transfusional IO and LIC decreased (mean absolute change) by –10.9 Fe/g dw at 1 year (*p* < .001), with an overall mean relative change of –42.9%; by year 2, the corresponding reductions were –13.5% and –47.9%, respectively, and the treatment was overall well tolerated (Kohgo et al., 2015). Significant reductions in plasma LPI have also been observed with deferasirox in an open-label, prospective, single-arm study of patients with MDS or other anemias, ongoing transfusion requirements, and SF levels of at least 1,000 ng/mL (Kim et al., 2015). Across all patients in the study (N = 100), there were significant reductions in SF by 1 year (*p* = .004), and LPI levels fell from 0.24 at baseline to 0.03 μmol/L by 1 year (*p* = .036; Kim et al., 2015).

Another study has shown increased 8-hydroxy-20-deoxyguanosine (8-OHdG) level (a measure of oxidative DNA damage) in the nucleus of peripheral blood mononuclear cells from MDS patients, as compared with those from healthy volunteers (Kikuchi et al., 2012). Interestingly, 8-OHdG levels were significantly increased in transfusion-dependent patients (high SF) vs. transfusion-independent patients (low SF), and there was a significant drop in 8-OHdG levels in the former group after 3 months of deferasirox therapy (Kikuchi et al., 2012). The findings of these studies thus provide evidence (other than SF decreases) of significant reductions in iron burden and its measurable adverse effects (e.g., oxidative damage) with deferasirox therapy in MDS patients, and offer, at least in part, a physiologic rationale for the improvements in survival (e.g., US 22; Kikuchi et al., 2012; Lyons et al., 2017).

## SAFETY AND TOLERABILITY OF IRON CHELATION THERAPY

**Figure 4:**
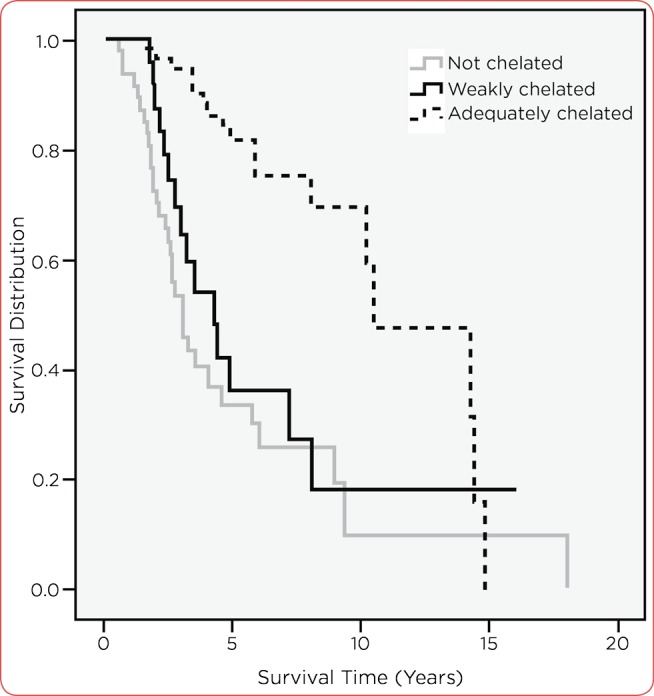
Overall survival among nonchelated (n = 47) vs. patients chelated > 6 months (n = 52). Reprinted from Delforge et al., Adequate Iron Chelation Therapy for at Least Six Months Improves Survival in Transfusion-Dependent Patients With Lower Risk Myelodysplastic Syndromes, Leukemia Research, 38(5), 557–563, Copyright © 2014, with permission from Elsevier.

The continuation of ICT for at least a 6-month period in transfusion-dependent patients with MDS has been recommended based on results showing improved survival with this duration of treatment (Figure 4; Delforge et al., 2014). In this study, the median overall survival was 3.1 years among the nonchelated patients vs. 10.5 years in those chelated for more than 6 months (*p* < .001); importantly, median survival was not different for those weakly chelated (fewer than 6 months) and those who were not chelated at all. The management of AEs associated with ICT and the degree of IO is therefore essential to achieve efficacy and benefit (Chaudhary & Pullarkat, 2013). A recent study showed that the effectiveness of deferasirox, measured by the decrease in SF (or LIC via MRI, if available), was significantly better in adherent patients (defined as those with a 90% or greater medication possession ratio) vs. nonadherent patients, and the median adherence rate in this small "real-world" sample (N = 35) was 92% (Escudero-Vilaplana, Garcia-Gonzalez, Osorio-Prendes, Romero-Jimenez, & Sanjurjo-Saez, 2016).

**Figure 5:**
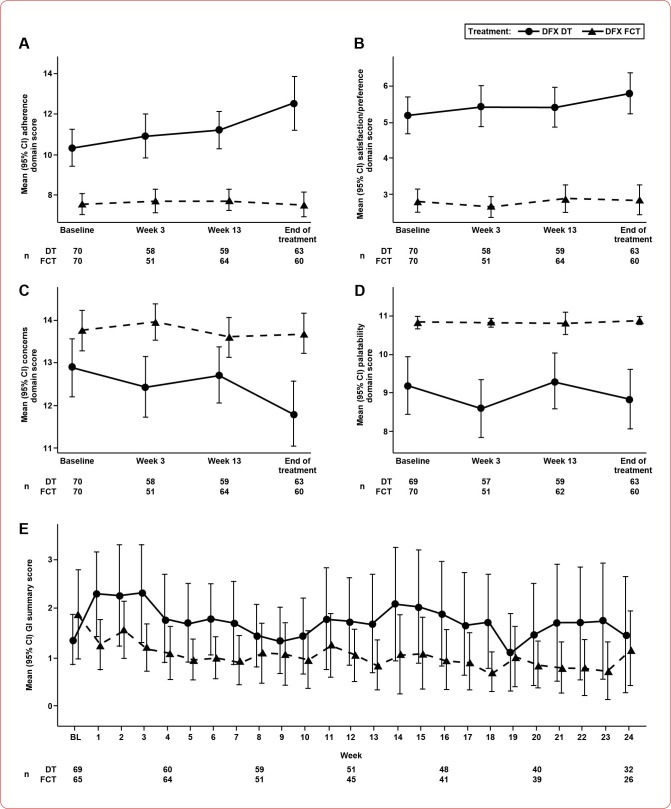
Mean domain scores for modified satisfaction with iron chelation therapy (A–C) and palatability (D) questionnaires over time by deferasirox treatment. (E) Mean (95% CI) gastrointestinal summary scores over time by deferasirox treatment. DFX = deferasirox; DT = dispersible tablet; FCT = film-coated tablet. Reprinted from Taher et al., American Journal of Hematology, 92(5), 420–428. Copyright © 2017 by Wiley Periodicals, Inc. Reprinted by permission of Wiley Periodicals, Inc.

The safety of deferasirox has been well defined across a range of clinical and "real-world" studies in MDS patients (Maurillo et al., 2015; Merkel & Nagler, 2014; Taran & Taran, 2015; Temraz et al., 2014). Gastrointestinal (GI) tract disturbances (nausea, vomiting, diarrhea, abdominal pain) are the most common and are generally mild and transient. Deferasirox may cause serious renal or hepatic toxicity, including failures or GI hemorrhage in certain patients. As such, therapy with deferasirox requires close patient monitoring, including laboratory tests of renal and hepatic function (Novartis Pharmaceuticals Corporation, 2016). Tolerability of oral agents (i.e., deferasirox and deferiprone) is also likely to be better than those requiring parenteral administration (e.g., deferoxamine). Recommendations for the management of GI AEs associated with deferasirox have been published (Nolte et al., 2015). The initial formulation of deferasirox was a dispersible tablet (DT) that had to be dispersed in water or juice prior to consumption; subsequently, a film-coated tablet (FCT) and the newest formulation, which is a sprinkle, were developed with a similar AE profile and better bioavailability (Nolte et al., 2015; Novartis Pharmaceuticals Corporation, 2017). Recently presented findings showed that patients treated with the DT had poorer adherence and satisfaction/preference vs. the FCT deferasirox formulation, and FCT-treated patients had fewer concerns and better tolerability (Figure 5; Taher et al., 2017). Patients on the FCT preparation of deferasirox also had better compliance and a longer continuation of treatment, allowing for greater SF reduction as compared with DT deferasirox (Taher et al., 2017).

## DISCUSSION

The pathophysiology of IO in MDS patients results from the underlying disease process (ineffective erythropoiesis, with attendant hepcidin suppression), genetic factors (e.g. *SF3B1*), as well as iron accumulation due to transfusion. Consequences of IO relate to iron deposition in tissues, accumulation of toxic iron species, and increased oxidative stress, all of which can potentially impact long-term outcomes, disease progression, and survival following ASCT. Due to its low cost and wide accessibility, SF will likely continue to be the most useful means to monitor IO in MDS patients. The bulk of available clinical data supports a benefit of using ICT in MDS patients with evidence of IO, particularly with deferasirox, the most widely studied iron chelator in this setting. Adverse events associated with deferasirox therapy have been largely manageable, allowing for effective durations of treatment, and the newer FCT formulation may allow for greater ease of administration for MDS patients. A general recommendation has been to initiate ICT when SF reaches greater than 1,000 ng/mL.

Further prospective evaluation of deferasirox in the TELESTO trial (ClinicalTrials.gov identifier: NCT00940602) is needed to confirm these data and further define the use of ICT in MDS patients (Shenoy et al., 2014; Taran & Taran, 2015). Life expectancy, transfusion burden, and evidence of iron excess (elevated SF), as well as any related patient comorbidities, are currently the main factors to consider when deciding on ICT in the absence of more rigorous evidence-based guidelines (Chaudhary & Pullarkat, 2013).
